# Perinatal Transmission of Dengue in a Neonate: A Case Report

**DOI:** 10.31729/jnma.8771

**Published:** 2024-10-31

**Authors:** Binita Chemjong, Luna Amatya, Binaya Raj Ghimire, Ganesh Dangal, Sonu Bharati, Sunita Maharajan

**Affiliations:** 1 Department of Pediatric Medicine, Kathmandu Model Hospital Institute of Health Sciences, Kathmandu, Nepal; 2 Department of Obstetrics and Gynecology, Kathmandu Model Hospital Institute of Health Sciences, Kathmandu, Nepal

**Keywords:** *case report*, *dengue*, *neonate*, *Nepal*, *pregnancy*

## Abstract

Vertical transmission of dengue is rare. However, the rapid rise of dengue infection is a risk in pregnancy which can lead to preterm delivery, low birth weight, stillbirth, miscarriage, neonatal morbidity and mortality. Here, we present a case of perinatal transmission of dengue in a term neonate who presented with fever on second day of life and desaturation without respiratory distress. Laboratory findings showed thrombocytopenia, dengue antigen NS1/IgM positive and treatment was started. There was positive maternal history of dengue 10 days prior to delivery. Hence, early investigations with prompt interventions was done leading to uneventful recovery.

## INTRODUCTION

Dengue virus is transmitted by female mosquitoes Aedes aegypti and the clinical symptoms of dengue can range from mild, non-specific acute febrile illness to severe life-threatening disease dengue hemorrhagic fever (DHF) and dengue shock syndrome (DSS).^1^ The dengue virus can affect all age groups. Dengue in pregnancy directly affects baby due to vertical transmission of virus.^2^ Dengue in neonate may manifest with fever, rash and thrombocytopenia and severe disease (DHF/DSS).^3^ Herein, we present a rare case of perinatal transmission of dengue virus in a term neonate born to a dengue positive mother at our center.

## CASE REPORT

A term (38+5 weeks) female, birth weight of 3.1kg with APGAR score of 6/10, 7/10 at 1^st^ and 5^th^ minute of life respectively was born to a 26 year primigravida through emergency lower segment cesarean section for meconium stained liquor in latent phase of labour. Mother had history of fever 10 days prior to delivery with dengue Ag NS1/IgM positive without warning signs and was managed symptomatically.

After delivery baby presented with desaturation (SpO2 88% in room air) without respiratory distress signs for which she was managed with oxygen supplement via head box in neonatal intensice care unit (NICU). Septic screening was sent to rule out neonatal sepsis which showed platelet counts of 139,000/cumm hence, baby was started on intravenous ampicillin and amikacin and contninued upto 7 days according to the hospital protocol. On 2^nd^ day of life baby developed multiple spikes of fever with maximum recorded temperature of 100.8 degree farenheit) which lasted for 5 day of life. She was able to maintain saturation and oxygen was weaned. On 3^rd^ day of life her platelets count decreased to 80,000/cumm. Vertical transmission of dengue was suspected due to unusual clinical presentation of fever and history of maternal dengue 10 days prior to delivery.

The laboratory findings of baby showed dengue antigen NS1/IgM positive and IgG negative on day 3 of life. Acute dengue fever was confirmed and intravenous fluid was started with close observation in NICU. Pathological and systemic examinations were unremarkable. C-reactive protein, coagulation profile, renal and liver function tests were within normal range. Her chest X-ray, abdominal and cranial ultrasound reports were normal. Blood culture showed no bacterial growth. Baby's platelets count gradually increased to 221,000/cumm on 8 day of life with stable hemodynamic parameters. She had uneventful recovery and was discharged on 8 day of life.

During her first follow-up after one week, baby was in healthy condition with no new fresh complaints.

## DISCUSSION

Vertical transmission is rare and is being reported globally. There is high risk of vertical transmission to the baby from mother resulting in perinatal morbidity and mortality.^4^ The increased level of viremia in pregnant women near or at delivery results in perinatal infection. The dengue infection in neonate can be asymptomatic or may manifest wide range of clinical features.^5^

Dengue is diagnosed with antigen NS1, virus isolation, nucleic acid detection or by checking antibodies in the first week of illness. Sensitivity depends on time the test is conducted and duration of illness. Dengue antigen NS1 level peaks within the first week of onset of symptoms with IgM reaching it's highest titer on 3-5 days of illness. The sharp rise in IgM antibody titer with dengue antigen NS1 positive is diagnostic of acute dengue infection.^1^ Following the incubation period 3-10 days, the onset of illness is rapid. In our case study, antigen NS1 and IgM tested positive on 3^rd^ day of life. Initially, perinatal transmission was not suspected thus virus isolation or antibody detection was not carried out in cord blood or placenta.

There are case studies reported which demonstrates maternal dengue at or near delivery resulting in perinatal transmission.^6-10^ In our case study, the mother was diagnosed with dengue fever 10 days prior to delivery and was managed conservatively. Jain A et al study has reported, infants with perinatal dengue infection can manifest mild fever, rash, thrombocytopenia and in severe cases hemorrhage, hepatomegaly, pleural effusion, ascites and mortality.^2^ In contrast, our patient presented with transient hypoxia, fever and thrombocytopenia which was also observed in studies reported.^7^ The pathological and systemic findings were unremarkable. In our case study, the possibility of perinatal transmission was significant with maternal dengue at late pregnancy, clinical symptoms and laboratory findings. Hence, early investigation, intervention and close monitoring lead both mother and infant to uneventful recovery.

Therefore, we conclude, this case report of perinatal transmission of dengue from Nepal, must bring awareness among healthcare providers and public health. Furthermore, physicians should be suspicious of perinatal transmission in maternal dengue and commence early investigation while ruling out neonatal sepsis. It is vital to closely observe the neonate till first two weeks of life to avoid the adverse complications. Research and studies should focus in creating neonate-specific dengue guidelines for best outcomes.
